# Method for removal of samples from the external ear canal and the tympanic membrane for histological and immunohistochemical studies

**DOI:** 10.1590/S1808-86942012000100006

**Published:** 2015-10-20

**Authors:** João Daniel Caliman e Gurgel, Celina Siqueira Barbosa Pereira, José Humberto Tavares Guerreiro Fregnani, Fernando de Andrade Quintanilha Ribeiro

**Affiliations:** a PhD Student in Medicine (Otorhinolaryngology) – School of Medical Sciences of the Santa Casa de São Paulo (ENT and Maxillo-facial surgeon); b PhD in Medicine (ENT) - School of Medical Sciences of the Santa Casa de São Paulo (Assistant Professor of the Morphology Department of the School of Medical Sciences of the Santa Casa de São Paulo); c PhD in Oncology - Antônio Prudente Foundation (Coordinator of the Researcher Support Group of the Barretos Cancer Hospital - PIO XII Foundation, Brazil); d PhD in Medicine (Otorhinolaryngology) – Paulista School of Medicine (Adjunct Professor of the Morphology Department of the School of Medical Sciences of the Santa Casa de São Paulo). Faculdade de Ciências Médicas da Santa Casa de São Paulo.

**Keywords:** ear canal, histology, immunohistochemistry, temporal bone, tympanic membrane

## Abstract

Temporal bones are valuable resources to study ear diseases. Although there are several methods for removing temporal bones from cadavers, such methods are not usually described in enough details in experimental research papers.

**Objectives:**

To describe a simple and rapid method for ear canal and tympanic membrane removal, and to evaluate its viability for histologic and immunohistochemical studies.

**Materials and Methods:**

In this experimental study, we obtained 31 ear canal and tympanic membrane samples from cadavers, with a conventional power drill and plug cutter. The material was dissected and samples containing ear canals and tympanic membranes were obtained in blocks. The samples were analyzed by histology and immunohistochemistry.

**Results:**

Removal of small and good quality samples containing entire ear canals and tympanic membranes. In all the samples, it was possible to perform both histological and immunohistochemical analyses.

**Conclusion:**

This method was easily achievable, reproducible and yielded good quality samples, both for training purposes and for experimental research. All the samples were viable for histological and immunohistochemical analyses.

## INTRODUCTION

Human temporal bones are valuable resources for the study of ear-related diseases. The pathophysiology of hearing loss, balance disorders, facial paralysis, ear disorders - such as cholesteatoma, among others, can be investigated through the study of temporal bone samples obtained from cadavers. These specimens are of extreme importance not only for experimental studies, but also for training physicians^1^, ^2^. In any medical training and residency program in Otolaryngology, it is extremely important to have temporal bones for study^3^. Despite the various methods available for obtaining temporal bone samples from cadavers, these methods are usually not described in details by the authors of the papers involving immunohistochemistry or experimental research histology^4^, ^5^, ^6^. During removal of the temporal bone from the body, special care must be taken to minimize autolysis and artifacts, which could impair the quality of the sample for studies. Factors such as: a history of sepsis, use of medication, prolonged death time, improper handling and fixating of the specimen, can interfere with cellular integrity, histological and immunohistochemical features, creating biases and hurting result analysis^1^.

When the goal of a particular paper involves analyzing only the epidermis of the ear canal and the tympanic membrane, the entire temporal bone is not necessary. In this case, it is possible to use a quick method that produces a smaller specimen that can be easily stored and transported in small containers.

The goals of the present study were to describe a convenient and fast method for obtaining samples of the external ear canal and tympanic membrane, and to assess its feasibility for histological and immunohistochemical studies.

## MATERIALS AND METHODS

This study's Project was submitted to the Ethics in Research Committee of the Institution, and it was approved under protocol # 052/10.

In order to carry out this observational study, we obtained 31 samples of the external acoustic meatus and tympanic membrane of cadavers.

The inclusion criteria were:

• Cadavers of persons who were victims of violent death;
• Less than 6 hours of death;

These criteria were used in the attempt to rule out cadavers with chronic diseases which could impact on the expression of immunohistochemical markers to be studied. There was a death time limitation so as to maintain cell integrity, thus enabling histological and immunohistochemical studies.

Exclusion criteria were:

• Temporal bone fracture with tympanic membrane and/or external acoustic meatus skin laceration;
• Cadavers with a history of hospital care immediately before death;
• Signs of systemic or ear infection;

The laceration of the ear canal skin or of the tympanic membrane could make it difficult to maintain the cylindrical shape of these structures during the paraffin block preparation. Other exclusion criteria were used to reduce the possibility of interference from drugs or infectious processes in the expression of the investigated immunohistochemical markers.

**Figure 1:**
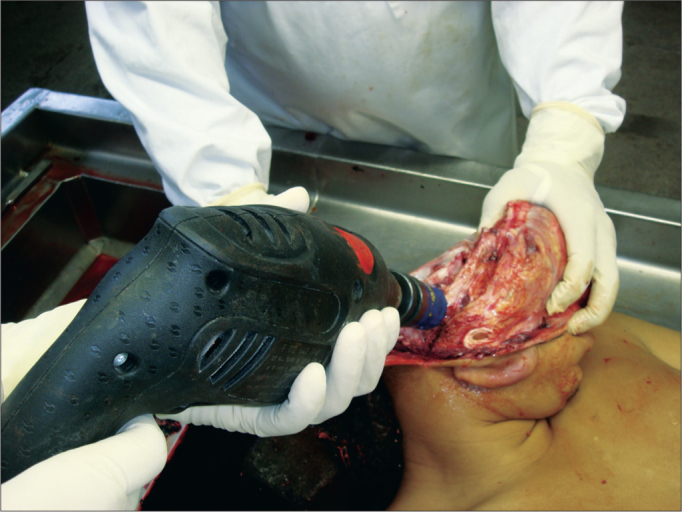
Drill and burr orientation to begin cutting.

**Figure 2:**
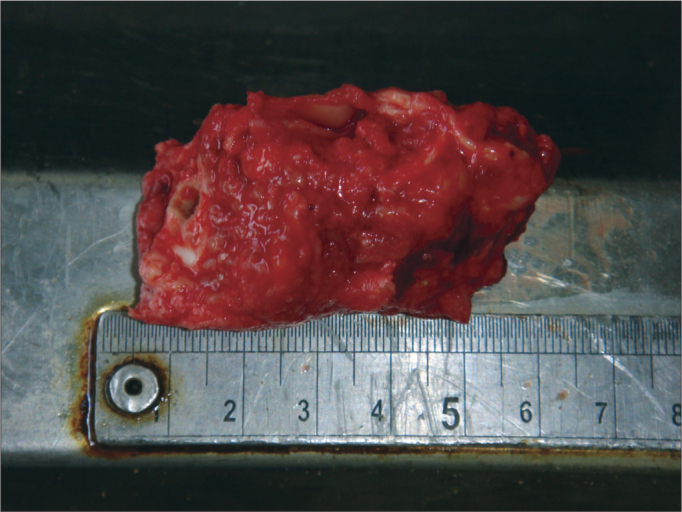
Specimen aspect after harvesting.

Obtaining samples began after conventional autopsy. The specimens were removed observing biosafety principles –wearing gowns, facial masks and gloves. After opening the skull and removing the brain, the temporal muscle was pushed and the ear canal opening was exposed as close as possible to the bone, facilitating the handling of the cup saw – of 5 cm in diameter and 6 cm deep. In order to start cutting, the activated saw was leaning on the surface to be removed, with little pressure on the bone in a mildly anterosuperior direction (Figure 1). With the cut already started, moderate pressure was employed until the end of the cutting process. Addressing the entire external auditory canal, tympanic membrane and middle ear, including the ossicular chain. After using the cup saw, with the aid of a 20mm chisel, an osteotomy was performed in the region of the petrous apex, releasing all bone attachments still remaining. The remaining muscle was incised with a scalpel and the specimen was finally removed (Figure 2). The fragment was immediately placed in 10% formalin. In the dissection lab, the fragment was placed in a proper support on a table with a microscope, vacuum, and the tools for dissection of the external ear canal and tympanic membrane.

**Figure 3:**
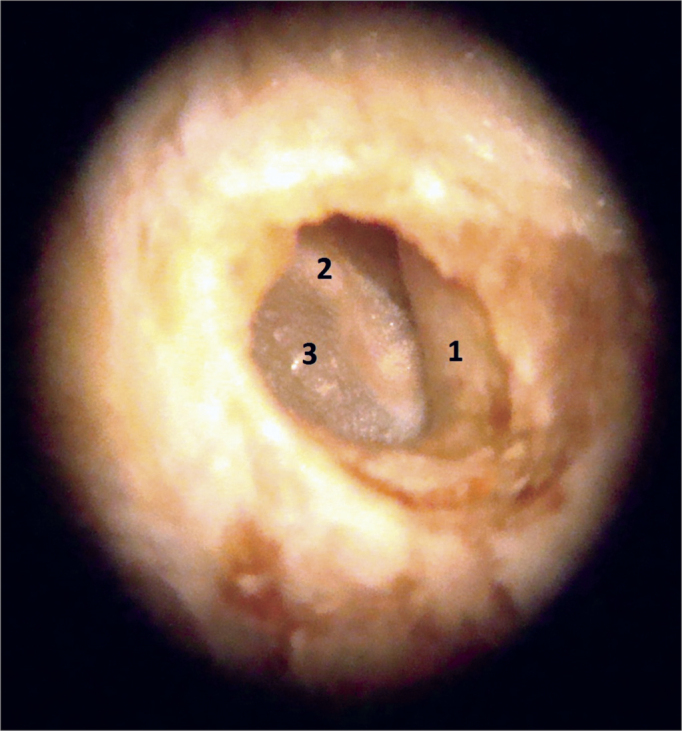
Specimen image under the microscope (10x magnification). 1: External acoustic meatus; 2: Malleus handle; 3: Tympanic membrane.

**Figure 4:**
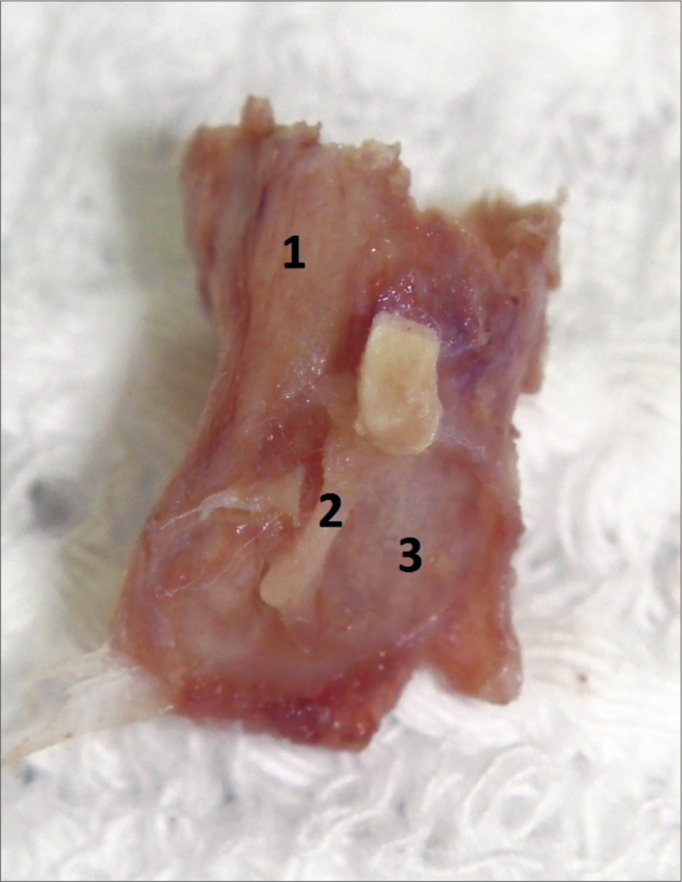
Final sample aspect, ready to be placed in a paraffin block. 1: External acoustic meatus; 2: Malleus handle inserted in the tympanic membrane mucosa; 3: Tympanic membrane mucosal membrane.

Before starting to use the microscope, yet remaining soft tissues were removed until close to the tympanic bone. The skin of the external auditory canal was completely dissected, always taking care to avoid lacerations, which could adversely affect the maintenance of the canal's cylindrical shape during the paraffin blocks preparation. The dissection and fibrocartilaginous annulus and tympanic membrane were always started posteriorly, as in a conventional tympanoplasty, and then extended to the entire circumference of the fibrocartilaginous annulus (Figure 3). The malleus was kept close to the tympanic membrane in order to facilitate the anteroposterior and superoinferior orientation of the specimen during the preparation of the paraffin blocks (Figure 4). In order to maintain the cylindrical shape of the ear canal close to the tympanic membrane, the paraffin was initially placed inside the canal and then around it, to make the blocks. The time between obtaining the samples and the preparation of paraffin blocks ranged between five and 15 days.

In order to test the feasibility of the method, we performed histological and immunohistochemical studies of the samples. The blocks were subjected to longitudinal sections in a rotary microtome, resulting in thee micrometer-thickness cuts for histological examination with hematoxylin-eosin (HE). The slides with fragments of the external ear canal and tympanic membrane were analyzed under the Axioscope 40 (Carl Zeiss from Brazil - optical microscope, with a 10x eyepiece and 5x, 10x, 20x and 40x objectives. The slides were photographed with an AxioCam MRC5 camera (Zeiss) connected to a computer. To obtain digital images of the material and area measurements, we used the Axiovision 4.8 software. The prepared slides contained the upper and lower walls of the meatus and the tympanic membrane in its largest diameter.

For immunohistochemical staining we used anti-CK16and Ki-67. Unstained sections in slides previously dipped in the adhesive organosilane underwent immunohistochemistry by immunoperoxidase procedure in three steps, with amplification by the avidin-biotin-peroxidase system without previous trypsinization^7^. Primary monoclonal antibodies used were the anti-cytokeratin 16 clone LL025 (DiagnosticBiosystems^®^, USA) and the anti-Ki-67 clone Ki-S5 (Dako^®^, Denmark), both at 1:100 dilution. The secondary antibody was the Max Polymer Detection System Kit (Novolink, Novocastra^®^) and the developer was diaminobenzidine (DAB, Dako^®^).

The slides were then evaluated by two experts on maintaining the epidermal histological and immunohistochemical (CK16 and Ki-67 expression) characteristics of the samples obtained by the method hereby described.

## RESULTS

**Figure 5:**
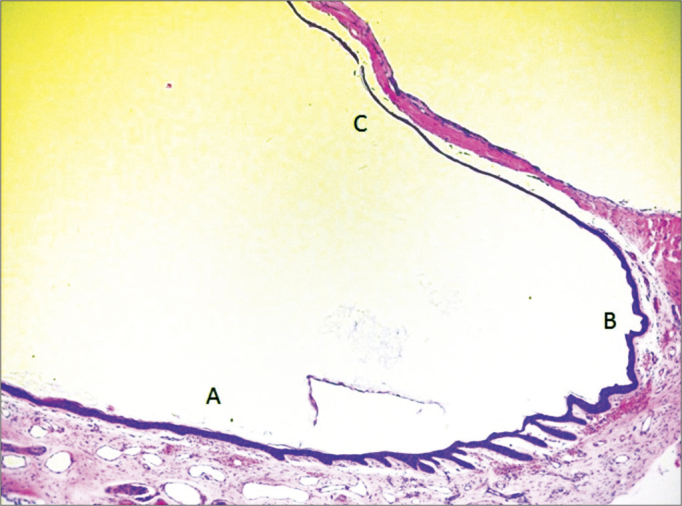
Micro-photography showing the epithelium of the lower portion of the external acoustic meatus (A), fibro-cartillagenous annulus (B) and tympanic membrane (C) (HE – 50x). Notice the presence of epithelial cones in the lower wall epidermis of the external acoustic meatus, near the fibro-cartilaginous annulus.

**Figure 6:**
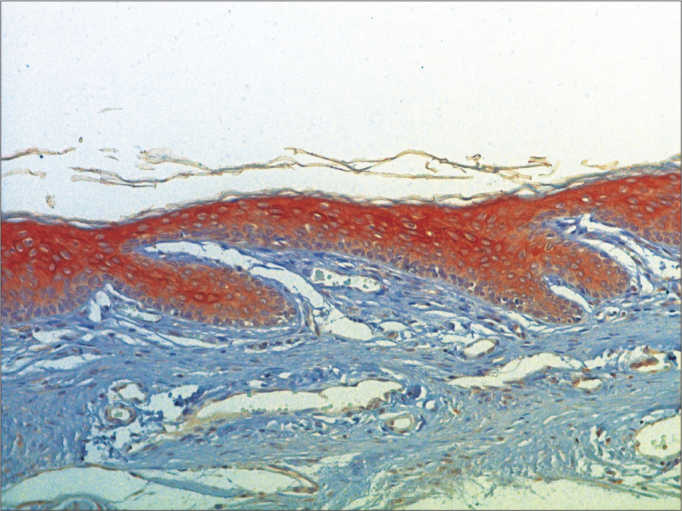
Micro-photography, showing the lower wall epidermis of the external acoustic meatus with CK16 expression (IHQ–200x).

**Figure 7:**
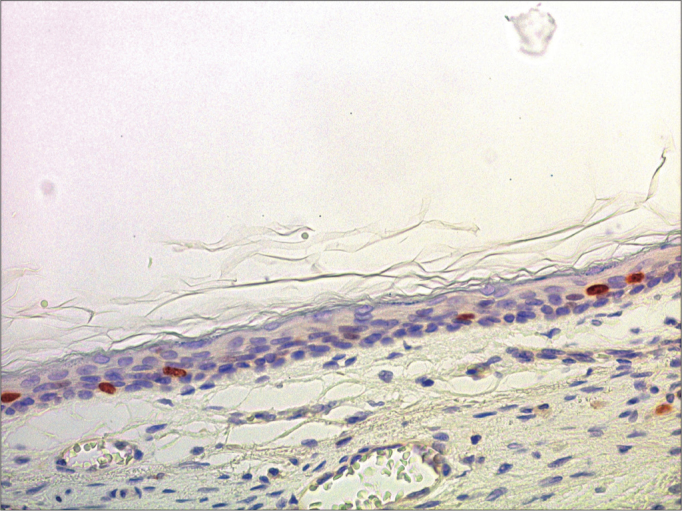
Micro-photography showing the lower wall of the external acoustic meatus with nuclei stained for Ki-67 in the basal layer and in the deeper portion of the spinnous layer (IHQ – 400x).

With improvements and training in using the technique, the time taken at each stage to obtain the samples was gradually shortened - according to the learning curve. The cadaver work phase initially lasted 30 minutes for each sample, before the technique was fully standardized. Once that happened, the fragments were obtained in just 2 minutes. During the dissection lab work, each specimen was initially dissected in 50 minutes. After the first dissections of the ear canal and tympanic membrane, only 15 minutes were needed for removal of the block samples without tearing the entire external auditory canal, the tympanic membrane and malleus for histological and immunohistochemical studies. All samples were viable for histological and immunohistochemical studies using this method (Figure 5, 6 and 7).

## DISCUSSION

Several methods have been described for obtaining samples of temporal bones for training purposes and experimental studies. To obtain samples of the temporal bone, its removal can be performed in a block, or to obtain more specific samples, cup saws can be used. The block removal can be accomplished by means of cuts with handsaws, power saws or with the aid of chisels. In this case, all the temporal bone was removed in a block (block method) and the bone can be studied in its entirety. Obtaining samples using cup saws can either be initiated on the inner surface of the skull, to obtain samples of the inner ear, including the internal auditory canal, but only for the medial region of the external ear canal, part of the mastoid cells, sigmoid sinus and only half side of the auditory tube^1^, ^3^. In the sideways method, the same as described in this paper, it is possible to obtain samples containing the entire external auditory canal, tympanic membrane, ossicular chain and part of the ear cavity^1^. Although several authors have already conducted experimental studies involving the external ear canal and the tympanic membrane from cadavers, few studies describe in detail the method for obtaining and preparing samples for histological and/or immunohistochemical studies^5^, ^6^, ^8^, ^9^.

The method hereby described, used low-cost tools (conventional drill and cup saws, used in carpentry) instead of special tools and saws as described before, obtaining similar results^1^. The instruments used in the dissection were similar to those used in other papers^8^. Published studies suggest the importance of the 24-hour time limit between material fixation and making the blocks, the need for injection of fixating material in the region to be studied or even cadaver cooling, to avoid artifacts or autolysis^1^. In this paper, these procedures were not required due to the short period between death and sample fixating in 10% formalin. The period between fixating and paraffin block making - between five and 15 days, did not have a negative influence on the quality of the samples, which was proven by histological and immunohistochemical studies.

## CONCLUSION

The method hereby described was easily doable, reproducible and produced good quality samples to be studied, either for training or experimental research. As the samples were being obtained, with greater experience in the method, there was a substantial decrease in the time required for executing each step. All samples were eligible for histological and immunohistochemical studies.
